# Comparative Study on the Hydrogenation of Naphthalene over Both Al_2_O_3_-Supported Pd and NiMo Catalysts against a Novel LDH-Derived Ni-MMO-Supported Mo Catalyst

**DOI:** 10.1021/acsomega.1c03083

**Published:** 2021-07-19

**Authors:** Ryan M. Claydon, Luis A. Roman-Ramirez, Joseph Wood

**Affiliations:** School of Chemical Engineering, University of Birmingham, Edgbaston, Birmingham B15 2TT, U.K.

## Abstract

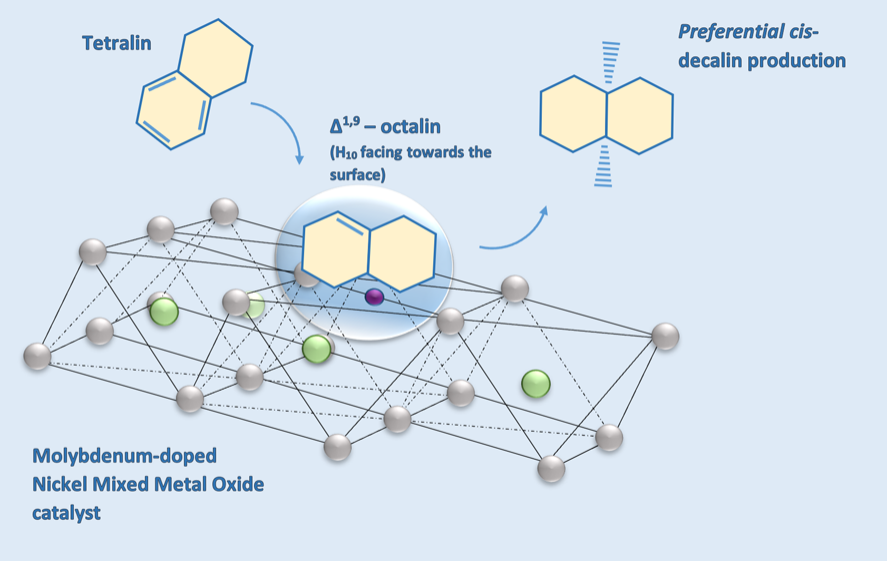

Naphthalene hydrogenation was studied over a novel Ni–Al-layered double hydroxide-derived Mo-doped mixed metal oxide (Mo-MMO), contrasted against bifunctional NiMo/Al_2_O_3_, and Pd-doped Al_2_O_3_ catalysts, the latter of which with Pd loadings of 1, 2, and 5 wt %. Reaction rate constants were derived from a pseudo-first-order kinetic pathway describing a two-step hydrogenation pathway to tetralin (*k*_1_) and decalin (*k*_2_). The Mo-MMO catalyst achieved comparable reaction rates to Pd_2%_/Al_2_O_3_ at double concentration. When using Pd_5%_/Al_2_O_3_, tetralin hydrogenation was favored over naphthalene hydrogenation culminating in a *k*_2_ value of 0.224 compared to a *k*_1_ value of 0.069. Ni- and Mo-based catalysts produced the most significant *cis*-decalin production, with Mo-MMO culminating at a cis/trans ratio of 0.62 as well as providing enhanced activity in naphthalene hydrogenation compared to NiMo/Al_2_O_3_. Consequently, Mo-MMO presents an opportunity to generate more alkyl naphthenes in subsequent hydrodecyclization reactions and therefore a higher cetane number in transport fuels. This is contrasted by a preferential production of *trans*-decalin observed when using all of the Al_2_O_3_-supported Pd catalysts, as a result of octalin intermediate orientations on the catalyst surface as a function of the electronic properties of Pd catalysts.

## 1 Introduction

Polyaromatic hydrocarbons (PAH) comprise the organic group containing two or more aromatic rings bonded together. These compounds represent a deleterious fraction in hydrocarbon fuels, leading to an incomplete combustion and consequently soot production, in addition to a reduction of the cetane number in diesel fuels.^1^ Environmental legislation is turning toward ever stricter guidelines that include reducing such compounds.^2^ These compounds are prevalent in lower-quality heavy oils. A unique conceptualization of the in situ combustion (ISC) process utilizes a horizontal well with catalyst pellets packed in the annular space. This is known as the toe-to-heel air injection and catalytic process in situ process (THAI-CAPRI),^3^ which has the potential to emanate refinery units such as primary and secondary stage hydrotreating reactors given the complex temperature zones up to 450 °C. This is particularly relevant at lower temperatures observed under the wet-mode ISC conditions in addition to thermal EOR mechanisms such as in situ upgrading technology (ISUT) which generates fields of temperature zones reaching 320 °C.^4,5^ Notwithstanding this, at the higher end of the temperature range, it has been shown by Chong et al.^6^ that monoaromatic and cycloalkane production is favored. However, identifying a material which could adequately refine or remove PAH compounds at a relatively price competitive margin promotes further investigation into the use of this technology.

Hydrogenation studies have previously focused on a great variety of catalytic materials. Noble metals have demonstrated the most significant rates of hydrogenation; however, the expense of the materials has led to the focus on transition metal-based catalysts.^7^ Many forms have been used in hydrogenation reactions including carbides, phosphides, oxides, nitrides, and sulfides.^8−10^

While PAH components in heavy oil typically involve very complex molecules, the use of a model compound such as naphthalene is useful to understand the selectivity and pathways governing polyaromatic group hydrogenation.^11^ Tetrahydronaphthalene (tetralin), a hydrogenated derivative of naphthalene, has been used previously to represent deleterious aromatics in fuel feeds.^12^ Hydrogenation of this molecule leads to the formation of both *cis*- and *trans*-decahydronaphthalene (decalin) via the intermediate pathway involving octahydronaphthalene (Δ^1,9^—octalin). These two products, however, comprise contrasting reactivities to ring opening and ring contraction.

*Cis*-decalin is far less thermodynamically stable, which results in greater selectivity to ring-opening reactions via selective hydrodecyclization.^13^ This conversion process results in the production of alkylated single-ring naphthenes from multi-ring naphthene precursors such as decalin, generating an improved cetane number.^14^ Consequently, this justifies the effort to maintain a high *cis-*/*trans*-decalin ratio. In previous works, the impact of tetralin concentration on the *cis/trans* ratio was shown to be negligible until most of the tetralin had undergone hydrogenation.^15^ Upon reaching the high conversion, the *cis/trans* ratio approached an equilibrium. It was suggested that the competition for catalytic adsorption sites was responsible for inhibiting *cis* to *trans* isomerization up until significant tetralin conversion. Wang et al.^16^ synthesized a chromium-based metal organic framework (MOF) for use in tetralin hydrogenation reactions spanning 140–220 °C across a range of 30–70 bar H_2_. The results indicated that a highly enriched cis/trans ratio was achieved across all levels of tetralin conversion, due to a greater adsorption facility for tetralin by the highly porous MOF support.

The choice of catalytic material is important as it can significantly affect the adsorption and dissociation energy barriers required for hydrogen activation and subsequent hydrogenation of the target molecule.^17^ Previous studies have examined the effect of hydrogen activation of individual Pd atom surface sites and the impact of particle size and dispersion on observed chemistry. A study conducted by Yu et al.^18^ summarized the beneficial impact of contiguous Pd active sites over a bimetallic catalyst, facilitating a greater dissociative adsorption of hydrogen over a bimetallic catalyst. It was suggested that contiguous Pd sites could be retained through either heat treatment or depositing a greater coverage of Pd. It has been found that when embedding Pd into Au, monomers are prevalent at lower coverages while an increased coverage generates clusters of Pd, which can have an impact on dissociation, spillover, and desorption of hydrogen.^17^

Many noble metal-based catalytic studies focus on second-stage hydrotreating (HDT) and hydrogenation (HYD) reactions deposited over protonic supports.^19−21^ This includes Al_2_O_3_-supported platinum and palladium catalysts.^15^ This electron deficiency, however, can result in overcracking and an overproduction of coke and catalyst deactivation. This can be mitigated using more neutral supports such as silica–alumina-supported noble metals or nonacidic supports.^22−26^ Works using supports such as zirconium-doped mesoporous silica have been used in the hydrogenation of tetralin, highlighting very active catalysis at 350 °C and 6.0 MPa hydrogen pressure.^27^ Research has also recently been undertaken to assess the impact of both noble and group VI metals over basic carriers in both HYD and hydrodesulfurization (HDS) reactions.^13,28−31^ It has been shown that a 2 wt % concentration of alkaline-earth metals dispersed over the conventional acidic support led to greater activity of platinum during the hydrogenation reaction of naphthalene.^13^ The works in Escobar et al.^13^ added to previous experimental work wherein basic supports were investigated and hypothesized to interact with metallic Ru nanoparticles, producing dual-site heterolytic hydrogen splitting and surface ionic hydrogenation pathways.^30^ Bimetallic Pd–Pt catalysts supported by Mg–Al mixed oxide were also synthesized to assess its activity for hydrogenation and hydrogenolysis of high-molecular-weight compounds as well as its thio-resistance, a necessary property given the presence of S-containing compounds in hydrotreated feeds.^32^ The results indicated that the catalyst exhibited high activity to decalin production; however, upon decreasing the Pd/Pt ratio, hydrogenation products decreased while the role of Pt-catalyzed hydrogenolysis reactions increased. Furthermore, hydrogenolysis reactions did not contribute to the formation of tar, which was in turn attributed to the lack of strong Bronsted acid sites exhibited by the basic mixed oxide support. Previous works have adopted a pseudo-first-order kinetic constant regime with respect to naphthalene concentration during the comparison of the catalysts.^13,26^

Previous studies^12,13,15,23^ have generated encouraging results which have prompted an assessment of whether a novel Mo-doped Ni-enriched mixed metal oxide (Mo-MMO) could provide an economical alternative to naphthalene hydrogenation, a compound used to represent deleterious compounds found in heavier oil reservoirs and thereby directed for refinement in first and second-stage HDT and HDA units. Layered double hydroxides (LDH)-derived mixed metal oxides (MMOs) have been largely studied in the literature in various reactions, owing to their tunability, resulting in variations in electronic configuration, dispersion and surface area, and reducibility, which have fundamental impacts on their performance as catalysts.^33,34^ Furthermore, the synthesis of LDHs remains an inexpensive alternative to typical highly tuned supports, due to the relative ease of coprecipitation and the ability to use a variety of waste streams for metal salt precursors.^35,36^ The MMOs that are obtained following LDH heat treatment generate a high surface area, a homogeneous solid solution of oxides which can have shown to be appropriate catalytic supports for HDT reactions.^37^

This study uniquely explores the possibility of using Ni-enriched, LDH-derived MMOs as supports doped with Mo using the incipient wetness impregnation technique. The reaction rate constant kinetic parameters from batch reactor experiments are constrained and contrasted against typical refinery grade Ni, Mo, and Pd-bearing catalysts on two-step naphthalene hydrogenation to tetralin and decalin. A comparison of *cis*-/*trans*-decalin ratios is made as a function of tetralin conversion during the hydrogenation reaction highlighting the apparent differences between the metal classes and both basic and acidic-enriched support materials introduced into the reaction. An assessment is made to understand how effectively catalysts can generate the compound, *cis*-decalin, which is more easily upgraded during further hydrodecyclization treatment.

## 2 Experimental Section

### 2.1 Catalyst Synthesis

A Ni-enriched LDH was synthesized using the coprecipitation method. Two metal salts, Ni(NO_3_)_2_·6H_2_O and Al(NO_3_)_3_·9H_2_O, were added to distilled water in the appropriate molar ratios to reach a 3.3:1 Ni/Al ratio in a 0.3 M 200 mL. The metal salt solution was sequentially pumped into a second solution containing sodium carbonate adhering to the appropriate ratio [CO_3_^2–^]/[M^3+^] = 0.5, to commence the in situ formulation of anionic clay. A NaOH solution was used to control the pH to promote LDH precipitation in a pH range of 9–10 and a stirring speed was set at 500 rpm. Precipitation occurred over the period of 12 h at 60 °C to promote adequate crystallization.

Following this precipitation and crystallization procedure, the resultant LDH was washed with deionized water to remove impurities. The samples were subsequently calcined at 450 °C for 4 h using a heating ramp rate of 10 °C min^–1^. Following this oxidation process, the resultant Ni-MMO was doped with Mo under incipient wetness impregnation in excess of toluene. A MoCl_5_ (anhydrous) salt was added in the appropriate ratio to achieve 10 wt % Mo. This method was carried out under constant stirring and a temperature of 60 °C for 12 h. Upon completion of the Mo-impregnation, the resultant Mo-doped MMO was calcined in air at a temperature of 450 °C for 4 h using a ramp rate of 10 °C min^–1^, to remove any remaining impurities.

Pd/Al_2_O_3_ catalysts are a preformulated series of industry catalysts containing 1, 2, and 5 wt % Pd, defined in previous studies.^38,39^

### 2.2 Catalyst Characterization

A Bruker D2 X-ray diffractometer (Co source and Ni filter) was used to generate Powder X-Ray Diffraction (PXRD) patterns. The method included a scan speed of 30 min with a step size of 0.370 over a 2θ range of 10–100° to determine the atomic arrangement of mineral crystals.

Changes to the catalyst structure as the temperature increased during calcination were analyzed using a NETZSCH TG 209 F1 to obtain a detailed thermogravimetric analysis (TGA). The method required 22 mg of catalyst set into a Pt crucible, with a heating rate of 10 K min^–1^ from 25 °C to the maximum temperature of 900 °C, under a constant flow set at 10 mL min^–1^ of air.

XRF was conducted on the sample using a S8 TIGER equipped with a high-intensity 4 kW Rhodium X-ray tube, two collimators at 0.23 and 0.46° and five analyzer crystals. Confirmation of the elemental constituents following impregnation of the molybdenum species was obtained.

Brunauer–Emmett–Teller (BET) analysis was performed using a Micromeritics ASAP2020 at the University of Warwick. To sufficiently evacuate the pores in the catalysts, the degassing procedure involved heating the samples to 350 °C and holding them for a duration of 6 h.

Temperature programmed desorption (TPD), performed by the University of Manchester, was used to evaluate the number of acid sites present in the catalyst. The method included loading 20 mg of the catalyst into a quartz U-tube reactor and analyzing the sample using a Quantachrome ChemBet Pulsar equipped with a thermal conductivity detector (TCD). The temperature ramp program included room temperature to 900 °C at a rate of 10 °C min^–1^ while simultaneously recording the intensity of NH_3_ 5% uptake in the He gas mix. Approximately, 40 mg of the catalyst was heated under He to 300 °C prior to holding for the duration of 1 h and subsequently cooling to room temperature. The NH_3_ 5% gas mix was then introduced for 2 h, followed by the replacement of the He gas, and a ramping program as before from room temperature to 900 °C under a 10 °C min^–1^ ramp rate.

The morphology of the catalysts was defined using transmission electron microscopy (TEM), while the distribution of the active metal species in the catalysts was mapped using a Hitachi TM3030 scanning electron microscopy (SEM) and energy-dispersive X-ray spectrometer (EDS).

### 2.3 Naphthalene Hydrogenation

The reaction was conducted in a 100 mL stainless steel Anton Parr batch reactor, conditions of which were chosen to reflect second-stage hydrotreating as used in previous work,^13^ at a fixed temperature of 250 °C, a stirring speed of 1000 rpm, a H_2_ atmosphere of 40 bar, and a catalyst to reactant ratio of 0.12/0.18 g, raising to 0.24/0.18 g with Ni and Mo catalysts, while the synthesized Mo-MMO catalyst was also used in a single loading 0.12/0.18 g to investigate the *cis*- and *trans*-decalin relationship. The stirring speed was selected to eliminate diffusion limitations prevalent in the three-phase heterogeneous reaction and therefore ensure the experiments were under direct kinetic control. During the 20 min heating-up stage, a N_2_ atmosphere was added to prevent any reaction prior to the reaction temperature set-point of 250 °C. At this temperature, a 0.5 mL vial was collected and analyzed to confirm the initial concentration of the reactants corresponded with the solution prior to heating. N_2_ was released and H_2_ at 40 bar was added to the reaction vessel. This was denoted as the time for reaction commencement. Liquor from the reaction was collected in vials at 20 min intervals for a 2 h period. The progression of the reaction was measured while the rate constants, *k*_1_ and *k*_2_ were derived reaction using a pseudo-first-order kinetic model. The pressure was maintained at 40 bar, and each vial collected represented a homogeneous solution of suspended catalyst, reactant, and product compounds at each time point.

All liquid samples were filtered using a micromembrane to remove catalyst particles, generating a clear liquid. The residual samples were then analyzed using Agilent Technologies 6890N GC with a corresponding 7683 B Series injector. An Agilent 19091J-413 capillary column (nominal length, diameter, and film thickness at 30.0 m, 320.0 μm, and 0.25 μm, respectively) was used accompanied with the following method: equilibration time of 3 min, with a ramp from 80 to 135 °C over 9 min and the second stage to 300 °C over 4 min. The components were separated according to boiling point. The identified reaction products included first- and second-stage hydrogenation products, tetralin, *cis*-decalin and *trans*-decalin, respectively. Five-point calibrations were made both isolated in the solvent and in the presence of the other products/reactants, to ensure no complications were prevalent in the analysis of reactant and product mixtures.

## 3 Results and Discussion

### 3.1 Catalyst Characterization

#### 3.1.1 LDH Thermal Degradation

**Figure 1:**
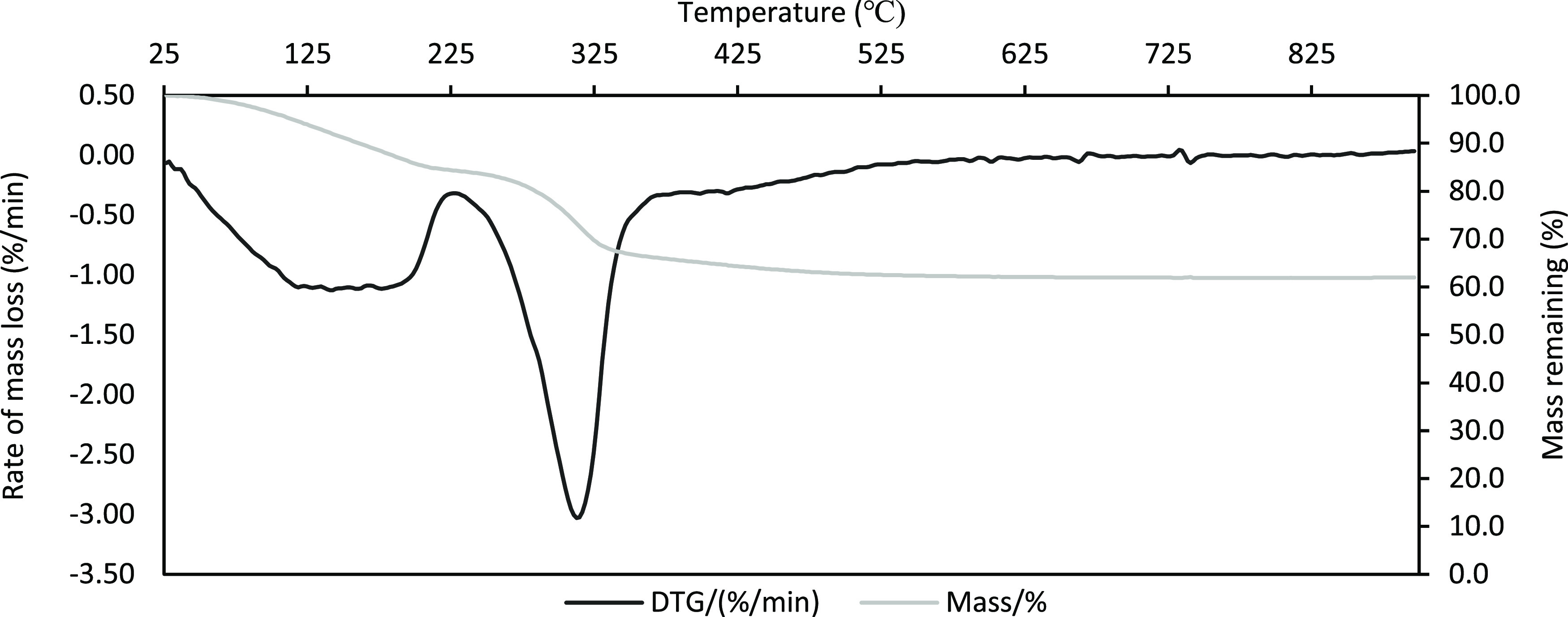
TGA profile of the nickel-enriched LDH, highlighting mass loss peaks corresponding to the structural properties of the material.

Thermogravimetric analysis of the synthesized nickel-enriched anionic clay highlights the process of MMO formation. The mass loss was measured as a function of time. Figure 1 highlights two distinctive peaks representing zones of mass loss which can be attributed to the loss of surface and interstitial water, in addition to interstitial decarboxylation and dehydroxylation of the metal hydroxide layers, respectively. This transition is in agreement with the literature,^40^ subsequently, resulting in a calcination process that produces a Ni-enriched MMO layer.

#### 3.1.2 Crystallographic Structure

**Figure 2:**
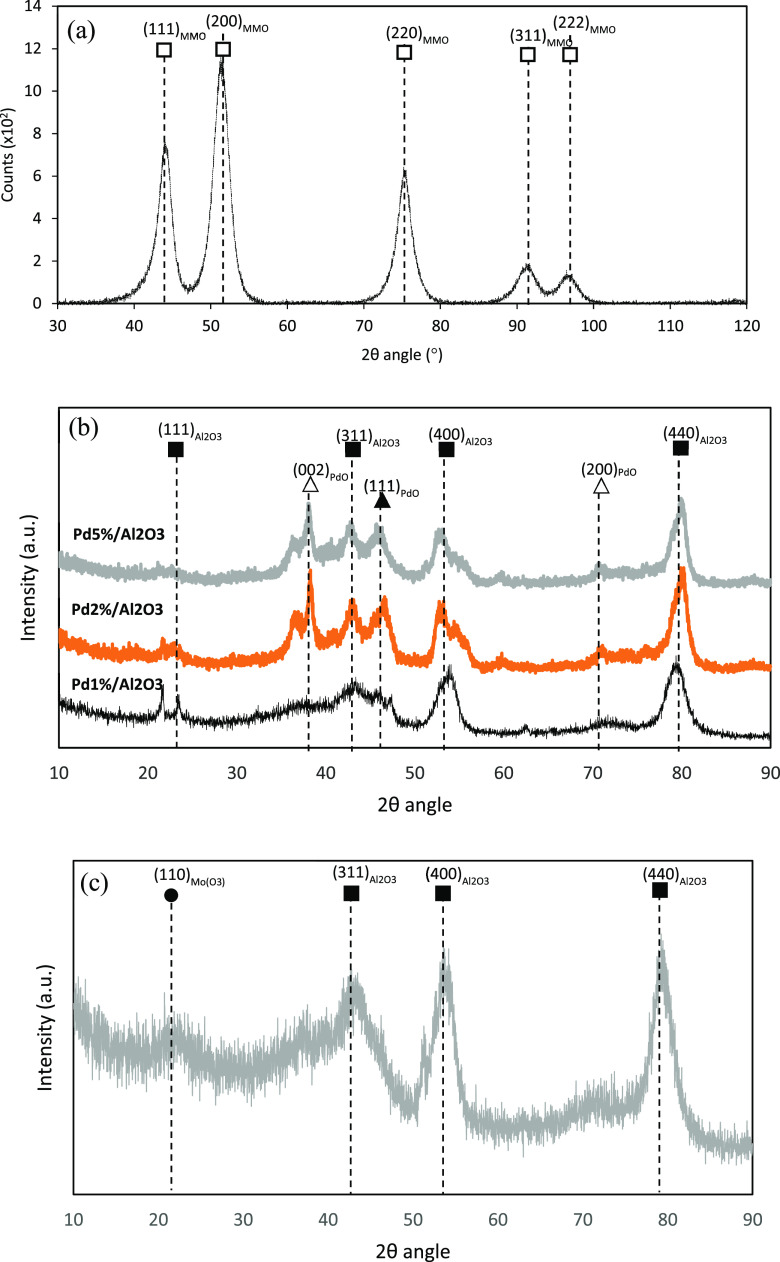
PXRD of (a) mixed oxides following calcination at 450 °C, (b) Pd_1%-5%_/Al_2_O_3_, and (c) NiMo/Al_2_O_3_; the peaks of which are correlated with 2θ angles depicted in the figures.

The Ni-enriched LDH was subject to a calcination process at 450 °C. This was to ensure that the material was completely delaminated forming high-surface-area polyphasic metal oxide sheets. The XRD pattern in Figure 2a highlights the residual components of the material detailing peaks matched to NiO, Bunsenite mineral as recorded on Powder Diffraction File database, no. 00-047-1049, with corresponding peak indices (111), (200), (220), (311), and (222), from low to high 2θ, respectively. It is assumed that while poor crystallization has led to the absence of aluminum in the XRD profile, both quasi-amorphous spinel type and Ni-doped Al_2_O_3_ phases can be found in the MMO.

The reflection peaks relating to Al_2_O_3_ and PdO phases, present in all of the alumina-supported Pd catalysts illustrated in Figure 2b, demonstrate the successive planes of (311), (400), and (440) and (002) and (200), respectively, reported by JCPDS no. 10-0425. The Pd_1%_/Al_2_O_3_ species, however, presents a poor indication of PdO peaks indexed at (002), (111), and (200), in agreement with the lower concentration of Pd deposited over the Al_2_O_3_ support.

When analyzing the XRD diffraction pattern for NiMo/Al_2_O_3_, it is evident that the crystallization of the material is poor. However, the Al_2_O_3_ peaks of (311), (400), and (440) are clear, while the MoO_3_ species present can be identified with the (110) peak. The nickel, however, is not observed due to its very small molar loading.

The XRF results confirm the presence of a nickel–aluminum mixed oxide in a ratio of 3.3:1, with a 9 wt % loading of Mo, while the refinery catalyst demonstrates an enrichment of aluminum relating to the acidic alumina support accompanied by surface nickel impregnation on the same order as the molybdenum (Table 1).

**Table 1:** Calculated Molar Ratios for Nickel and Molybdenum Catalysts Using XRF Analysis

	catalyst	
element	Mo-MMO	NiMo/Al_2_O_3_
Ni	1.10	0.07
Al	0.33	1.25
Mo	0.09	0.09

#### 3.1.3 Textural Properties

The surface texture measurements of the catalysts are highlighted in Table 2. It is clear that NiMo/Al_2_O_3_ demonstrates the highest surface area, coupled with the greatest pore volume and comparatively large pore size. When drawing comparisons with the LDH-derived catalyst, it is clear that the greater porosity and surface area observed over the NiMo/Al_2_O_3_ catalyst should theoretically provide for a higher activity in terms of upgrading larger components typically found in heavier oil feeds. When contrasting against the characterizations of Pd-based catalysts, it is evident that while Pd_5%_/Al_2_O_3_ is outperformed on pore volume and surface area, the average pore size is superior. This is a characteristic that will inhibit the coking of pore throats during the upgrading of heavier feeds.

**Table 2:** Textural Properties of the Catalysts Used in This Study, Derived from BET Analysis

	Mo-MMO	NiMo/Al_2_O_3_	Pd_1%_/Al_2_O_3_	Pd_2%_/Al_2_O_3_	Pd_5%_/Al_2_O_3_
pore volume (cc g^–1^)	0.39	0.67	0.48	0.63	0.38
surface area (m^2^ g^–1^)	141.62	220.58	186.88	119.15	110.37
average pore size (Å)	54.5	60.7	51.3	10.6	69.7

#### 3.1.4 Acidity

**Figure 3:**
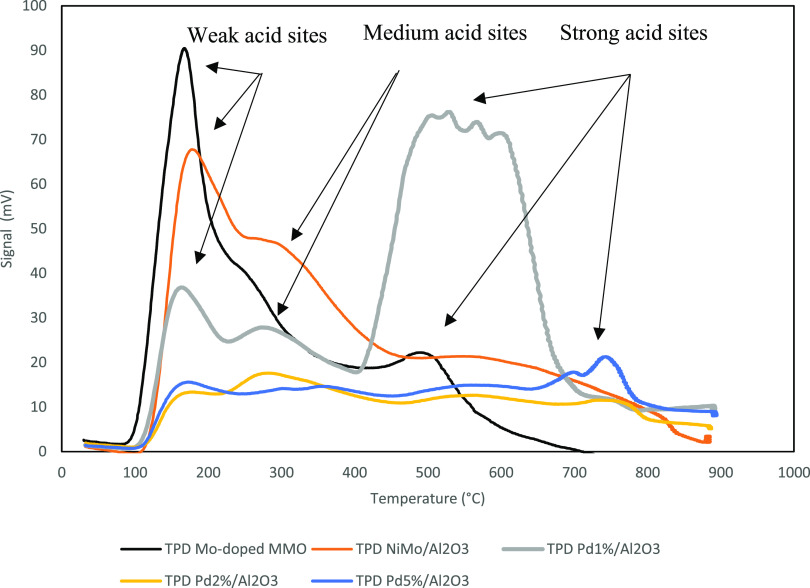
TPD profiles for the Mo-doped Ni-MMO species, NiMo/Al_2_O_3_ catalyst, and Pd_1%_–Pd_5%_/Al_2_O_3_.

The peaks of reduction and extent of NH_3_ desorption across the temperature range of 0–900 °C are shown in Figure 3, with the total acidity defined in Table 3.

**Table 3:** Acid Site Count Using TPD NH_3_

catalyst	acidity via NH_3_ desorption (mmol g^–1^)
MMO support	0.368
Mo-MMO	2.04
NiMo/Al_2_O_3_	2.64
Pd_1%_/Al_2_O_3_	2.79
Pd_2%_/Al_2_O_3_	0.84
Pd_5%_/Al_2_O_3_	1.01

NH_3_ (5%)-TPD was performed to investigate the acidic properties of the catalyst. The peaks can be separated into three predominant groups, representing the strength of the acid site. As temperature increases, the strength of the acid site increases, generating bands of weak, medium, and strong acid sites, the temperature ranges of which follow the approximation 100–200, 250–350, and 450 °C. The total number of acid sites has been calculated at 0.368 mmol g^–1^ for the Ni-MMO support and 2.04 mmol g^–1^ for the molybdenum-doped MMO. The Mo-doped MMO demonstrated a sharper peak with a signal of 89 mV, an order of magnitude higher than the support, at 168 °C. This was followed by a smaller secondary peak at 490 °C and ultimately indicates a significant presence of weaker acid sites which are active at lower temperatures following Mo-impregnation.

Comparatively, the NiMo/Al_2_O_3_ catalyst exhibited broader peaks over a greater temperature range indicating the presence of stronger acid sites. The Pd_1%_/Al_2_O_3_ material exhibited two smaller peaks at low temperatures, 163 and 273 °C, before reaching a more significant and broader peak between 510 and 610 °C, indicating the dominance of stronger Lewis acid sites. The Pd_2%_/Al_2_O_3_ and Pd_5%_/Al_2_O_3_ catalysts exhibited a low-intensity broad peak ranging from 100 to 900 °C, while the latter comprised a small peak at 744 °C. For comparison, a γ-alumina catalyst bearing molybdenum, promoted by cobalt, exhibits a total acid site count of 1.513 mmol g^–1^ when using NH_3_ as the adsorption agent.^41^

#### 3.1.5 Morphology and Metallic Distribution

**Figure 4:**
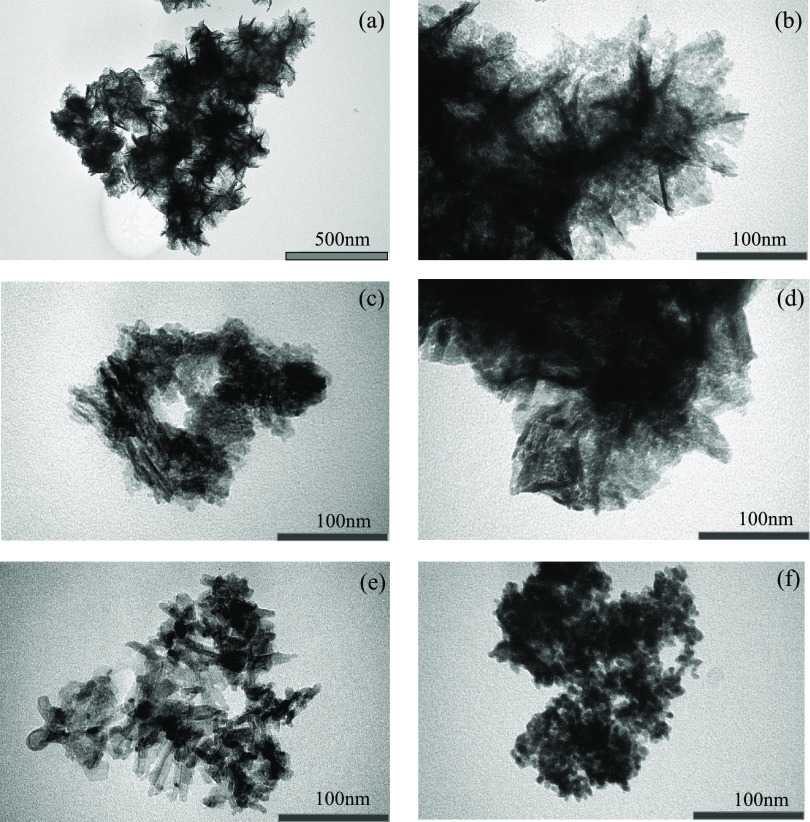
TEM imagery to elucidate the crystal structure and shape: (a, b) Mo-MMO, (c) NiMo/Al_2_O_3_, (d) Pd_1%_/Al_2_O_3_, (e) Pd_2%_/Al_2_O_3_, and (f) Pd_5%_/Al_2_O_3_.

The images in Figure 4a,b highlight the morphological structure of the heat-treated LDH structure. An approximation of the particle size pertains to approximately 200 nm crystallites. The plate-like agglomerates are typical of rhombohedral crystallites in LDH-derived materials. The NiMo/Al_2_O_3_ image, Figure 4c, demonstrated less well-defined tubular morphology, approximating 50 nm in length and 5 nm in transverse length.

TEM imaging in Figure 4e reveals that the Al_2_O_3_ product in Pd_2%_/Al_2_O_3_ consists of disordered stacking nanofibers of length concentrating around 100 nm, with a transverse width of less than 10 nm. The Pd_1%_/Al_2_O_3_ morphology, shown in Figure 4d, demonstrates very similar attributes, approximating the same dimensions. Pd_5%_/Al_2_O_3_ demonstrates a sphere morphology, the diameter of which does not generally exceed 10 nm, which can be observed in Figure 4f.

**Figure 5:**
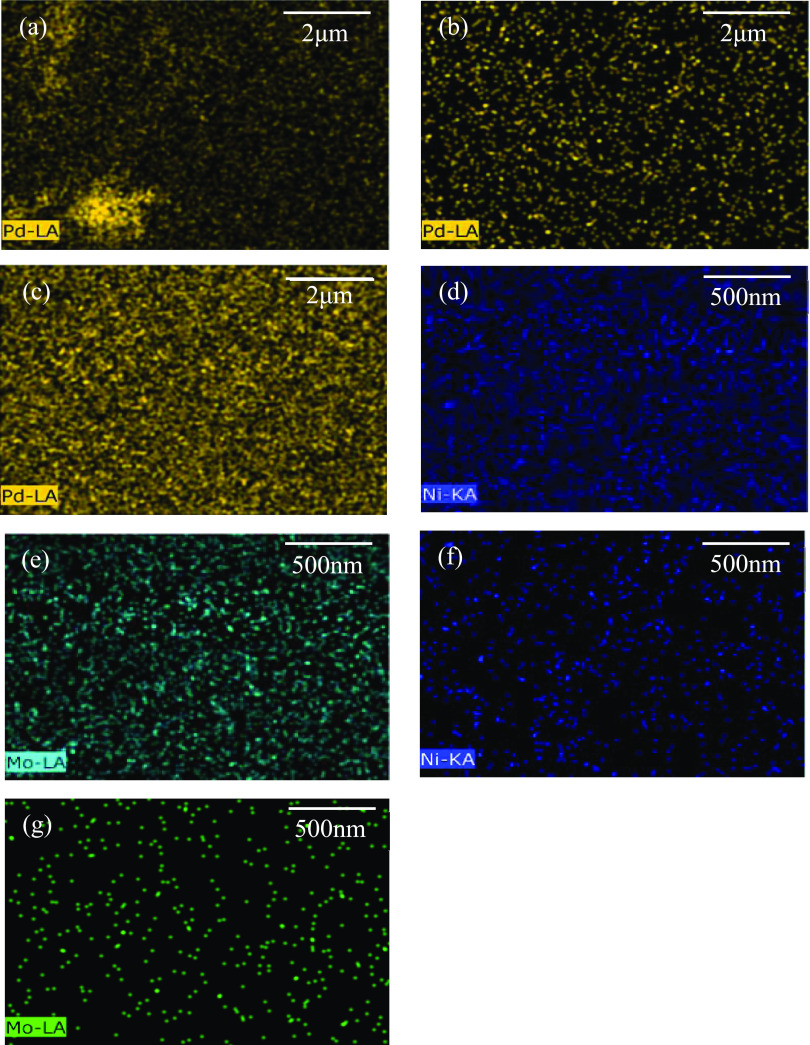
EDX analysis of Pd/Al_2_O_3_ catalysts highlighting the distribution of Pd over the catalysts as a function of wt % for (a) Pd_1%_/Al_2_O_3_, (b) Pd_2%_/Al_2_O_3_, and (c) Pd_5%_/Al_2_O_3_, in addition to the distribution of Ni and Mo over Mo-MMO (d, e) and NiMo/Al_2_O_3_ (f, g), respectively.

The EDS analysis of Pd/Al_2_O_3_ catalysts clearly indicates the increased concentration of Pd deposited over the Al_2_O_3_ support as the loading increases. With Pd_1%_/Al_2_O_3_ in Figure 5a, the dispersion is not homogenous, rather areas of highly dispersed and agglomerated Pd exist, which is observed as random bright spots occupying the frame. As the concentration increases to Pd_2%_, as observed in Figure 5b, Pd is more homogeneous in its dispersion with higher density Pd epicenters deposited across the support. As the concentration increases to Pd_5%_ in Figure 5c, it is clear that again even richer epicenters of contiguous Pd exist, while the dispersion of the Pd is such that only a small fraction of the frame is Pd-deficient. This correlation may be linked to the sequential reduction in surface area exhibited by the catalysts with the trend as follows Pd_1%_ > Pd_2%_ > Pd_5%_, demonstrated in Table 2.

The distribution of Ni and Mo over the Mo-MMO and NiMo/Al_2_O_3_ catalyst show that the textural properties of anionic clay-derived MMO and Al_2_O_3_ supports affect the dispersion of the active species significantly. With the Mo-MMO support, nickel is embedded as nickel oxides in a solid solution of both nickel and aluminum oxides making up the MMO. This leads to a more homogeneous distribution, as highlighted in Figure 5d. The NiMo/Al_2_O_3_ catalyst is limited by both the concentration of nickel embedded on the support, as the content of nickel in the catalyst is comparatively lower, as well as the nature of its deposition through impregnation, subsequent to the preparation of the alumina support. As a result, Figure 5f highlights the heterogeneous dispersion with a lower Ni signal.

There is a clear difference in Mo distribution between the catalysts. As shown in Figure 5e, the distribution of Mo is more homogeneous over the Mo-MMO catalyst while also exhibiting a comparatively higher signal. The heat-treated anionic-clay-derived MMOs form large high-surface-area planar oxide layers with a more limited pore network, as attested in Table 2. Consequently, it is expected that the Mo deposition is concentrated on the surface of the catalyst, while Mo incorporation into an Al_2_O_3_-supported catalyst, which is characterized by a high pore volume and much greater surface area at 220.56 m^2^ g^–1^, will generate a greater distribution of internal Mo active centers not observed by the EDS analysis, hence the distribution of isolated Mo centers in Figure 5g.

### 3.2 Kinetic Study

**Scheme 1:**
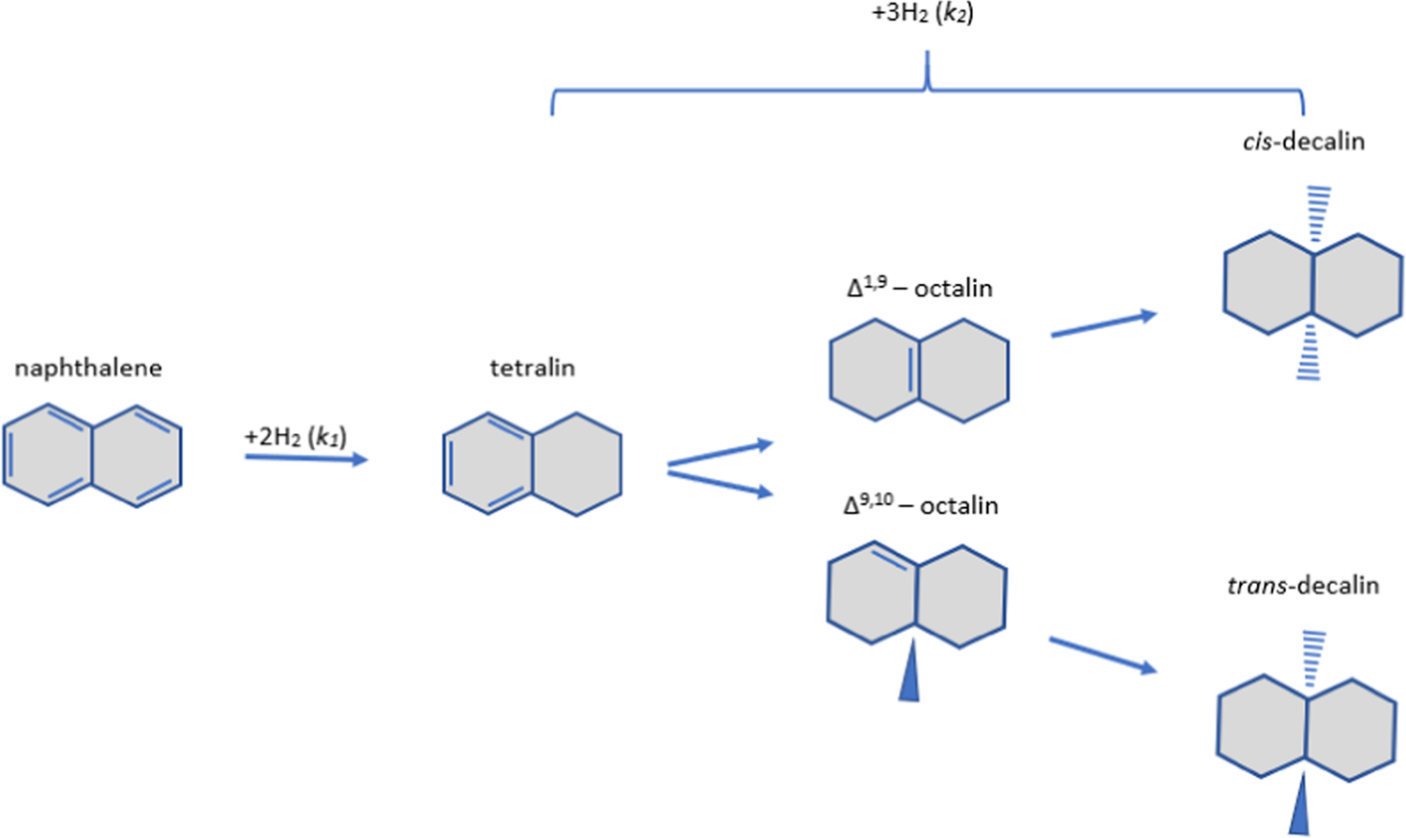
Naphthalene Hydrogenation Reaction Pathways

Naphthalene undergoes partial hydrogenation to tetralin before complete hydrogenation to decalin, according to Scheme 1.

Decalin, however, comprises two particular isomers, cis- and trans-, where cis- is typically favored for improving the cetane number. The pseudo-first-order model, used to derive values for *k*_1_ and *k*_2_, was selected as the enrichment of H_2_ comprising the sole component in the reaction gas (H_2_ ≫ Naphthalene) meant that it could be regarded as constant. Similar to previous works,^13^ the reaction mechanism can then be defined by the following set of equations
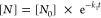
1
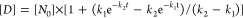
2
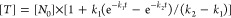
3where *N*, *D*, and *T* stand for the naphthalene, decalin, and tetralin concentrations, respectively, and *N*_0_ is the naphthalene initial concentration. The reaction rate coefficients *k*_1_ and *k*_2_, shown in Table 4, were obtained simultaneously by minimizing the objective function, sum of squares of residuals (SSR), between the experimental and model-calculated naphthalene, tetralin, and decalin concentration data points. The solver used was a nonlinear generalized reduced gradient (GRG) on Microsoft Excel.

**Table 4:** Calculated Reaction Rate Constants, Assuming Pseudo-First-Order Kinetics, for Naphthalene Conversion to Tetralin and Tetralin Conversion to Decalin, Denoted *k*_1_ and *k*_2_, for Each Catalyst Condition

	pseudo-first-order kinetic rate constant	
catalyst	*k*_1_ (min^–1^)	*k*_2_ (min^–1^)
Pd_5%_/Al_2_O_3_	0.0690	0.2240
Pd_2%_/Al_2_O_3_	0.1059	0.0031
Pd_1%_/Al_2_O_3_	0.0069	0.0012
NiMo/Al_2_O_3_	0.0036	0.0006
Mo-MMO	0.0353	0.0028

**Figure 6:**
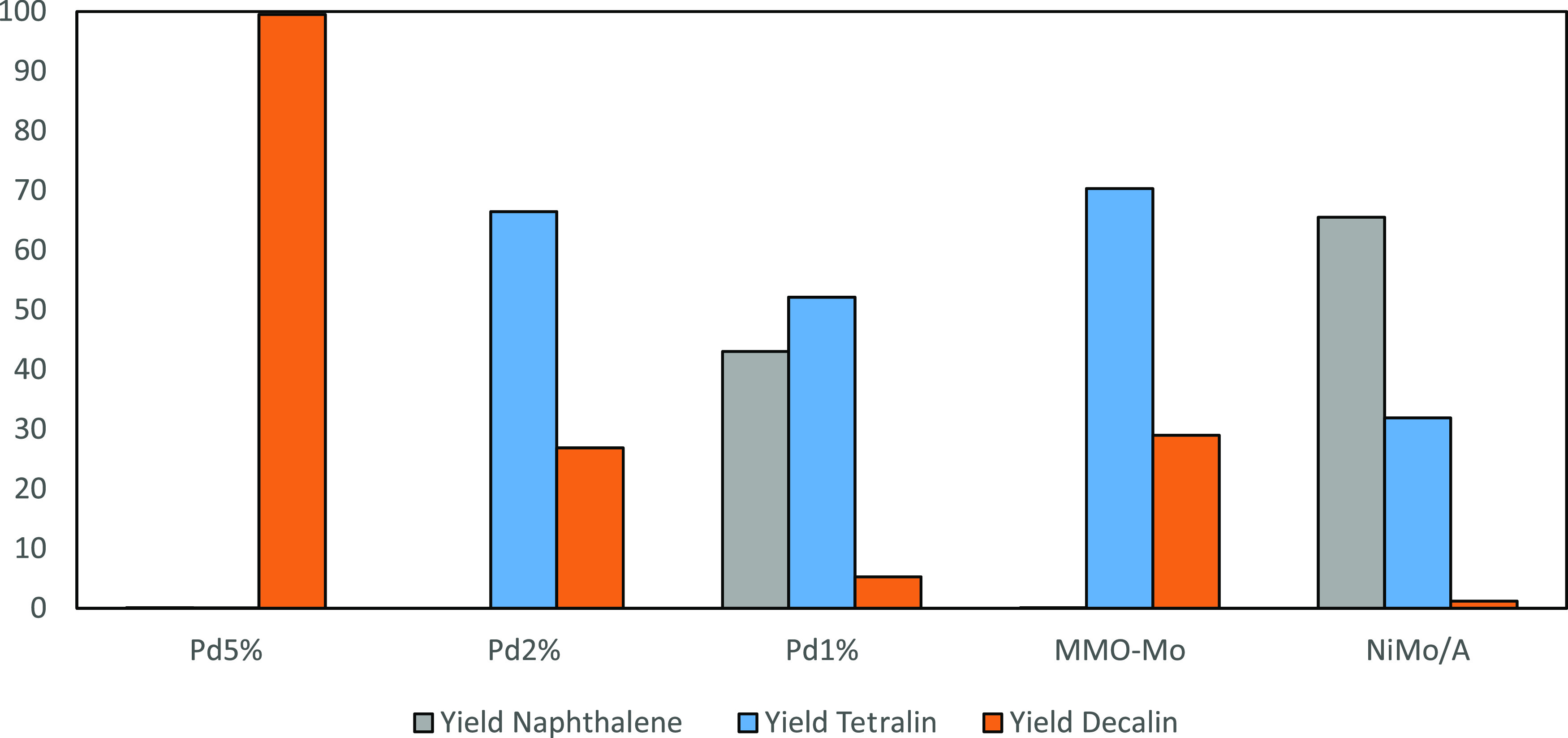
Yield of naphthalene, tetralin, and decalin (cis and trans) for each catalyst regime.

**Figure 7:**
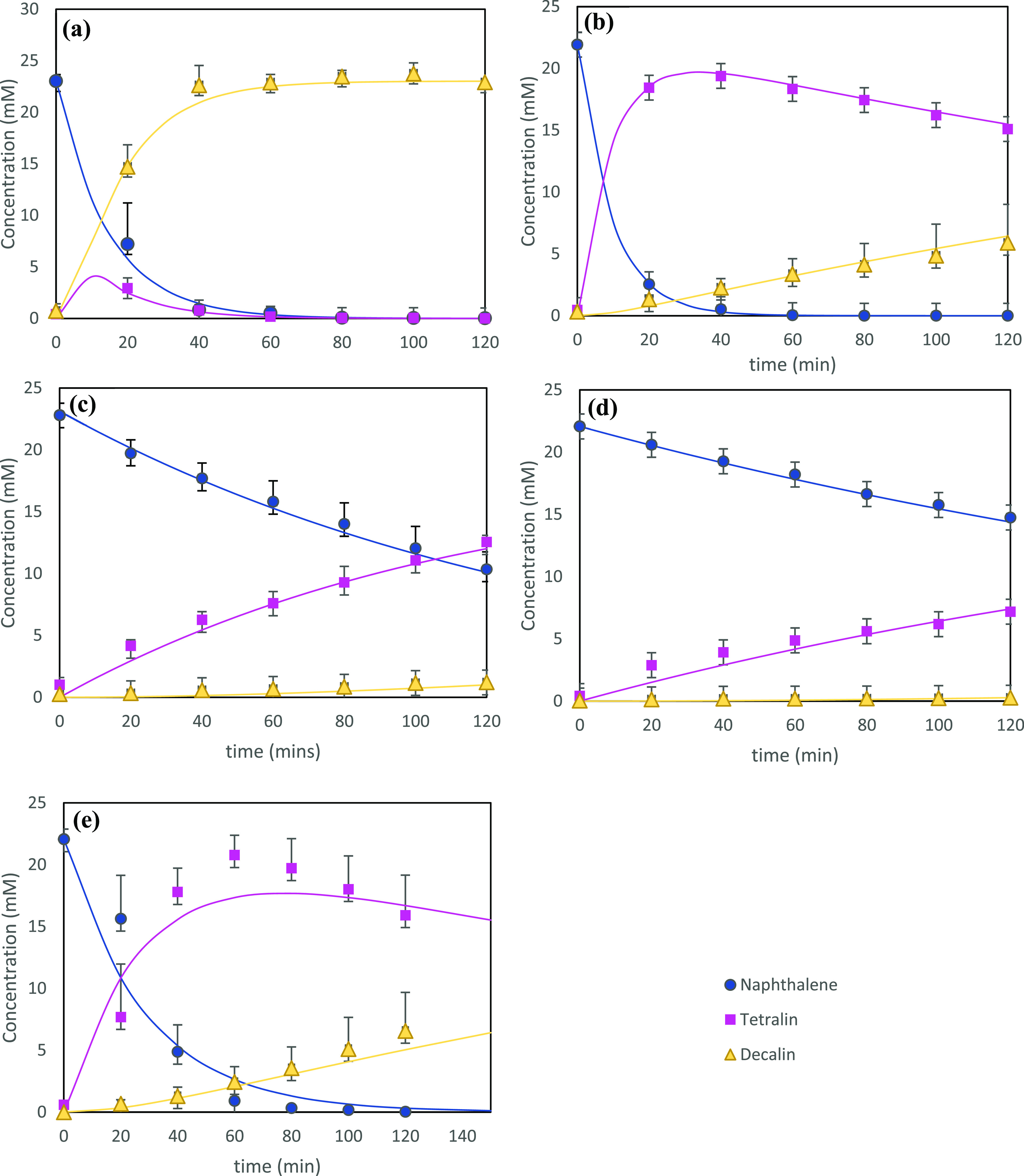
Plots of naphthalene, tetralin, and decalin concentration against time using the experimentally derived data and pseudo-first-order kinetic model for each catalyst: (a) Pd_5%_/alumina, (b) Pd_2%_/alumina, (c) Pd_1%_/alumina, (d) NiMo/alumina, and (e) Mo-MMO.

**Figure 8:**
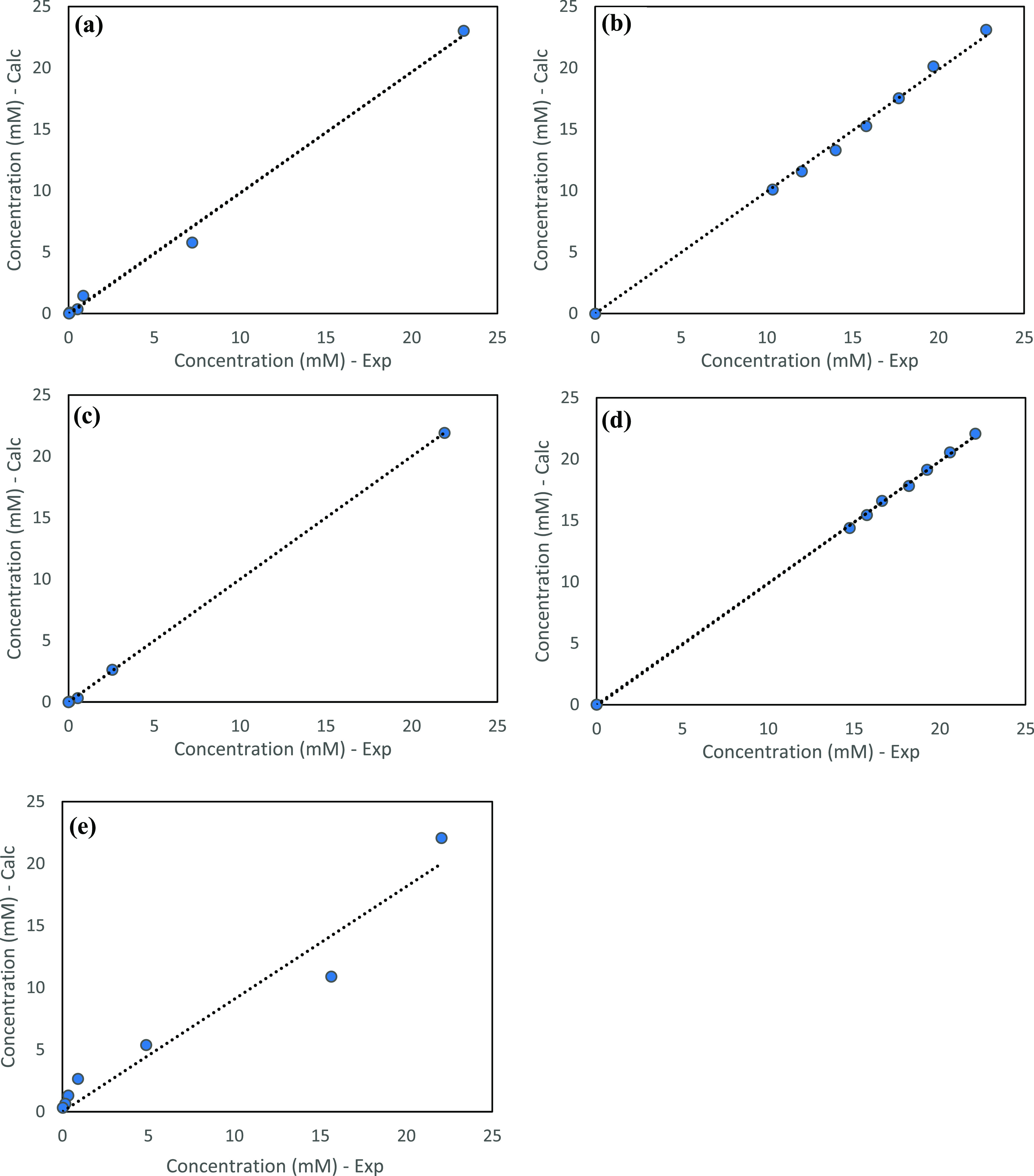
Parity plots for naphthalene concentration: (a) Pd5%/alumina, (b) Pd_2%_/alumina, (c) Pd_1%_/alumina, (d) NiMo/alumina, and (e) Mo-MMO.

The yields of naphthalene, tetralin, and decalin for each catalyst regime are demonstrated in Figure 6. The experimental and modeling results are presented graphically in Figure 7, while the corresponding parity plots are shown in Figure 8.

In terms of the catalytic activity as shown in Figure 6, the Pd_5%_/Al_2_O_3_ catalyst is significantly more active than any of the other catalysts in the hydrogenation of naphthalene as can be observed by the conversion. This can be attributed to both the comparatively high concentration of Pd deposited over the alumina as well as the presence of contiguous Pd active sites which promote dissociative H_2_ adsorption. Pd_5%_/Al_2_O_3_ achieves a yield of 99.5% decalin, while as the concentration of the Pd loading decreases over the alumina support, so does the total decalin yield, to 26.9 and 5.3% for Pd_2%_/Al_2_O_3_ and Pd_1%_/Al_2_O_3_, respectively. While Pd_1%_/Al_2_O_3_ demonstrates a significantly higher proportion of acid sites as a result of the increased exposure of alumina acid sites, correlating with the increased surface area at 186.66 m^2^ g^–1^, it is clear that the hydrogenation of naphthalene is limited by the inhibited hydrogen adsorption facility of the catalyst with the lower concentration of Pd. The yields of decalin for Ni and Mo-bearing catalysts are 29 and 1.2% for Mo-NiMMO and NiMo/Al_2_O_3_, respectively.

In terms of tetralin formation, the lowest yield follows the following trend: Pd_5%_/Al_2_O_3_ < Mo-NiMMO < Pd_2%_/Al_2_O_3_ < Pd_1%_/Al_2_O_3_ < NiMo/Al_2_O_3_. It is clear that NiMo/Al_2_O_3_ has the worst selectivity for hydrogenation products after the reaction time. The majority of the product is naphthalene, whereas for all of the other catalytic regimes, it is clear that either hydrogenation products, tetralin or decalin, are favored. The only catalyst with a clear selectivity toward decalin products is Pd_5%_/Al_2_O_3_.

When modeling according to pseudo-first-order kinetics, *k*_1_ and *k*_2_ values were determined as shown in Table 4. The model used is presented graphically in Figure 7 in relation to the experimental results. The order of *k*_1_ persists as follows: Pd_2%_/Al_2_O_3_ > Pd_5%_/Al_2_O_3_ > Mo-MMO > Pd_1%_/Al_2_O_3_ > NiMo/Al_2_O_3_. The order of *k*_2_ values persists as follows: Pd_5%_/Al_2_O_3_ > Pd_2%_/Al_2_O_3_ > Mo-MMO > Pd_1%_/Al_2_O_3_ > NiMo/Al_2_O_3_. As the Pd loading increases, it would be expected that *k*_1_ and *k*_2_ values would increase; however, the reduction of *k*_1_ values at a certain maximum contravenes this prediction.

When using Pd_5%_/Al_2_O_3_ coupled with this pseudo-first-order reaction model, it becomes clear that the assumption of the second step being the rate-determining step (*k*_1_ ≫ *k*_2_) does not hold. This is a detour from almost all conventional assumptions, which also makes the simplified form provided in Escobar et al.,^13^ used for the platinum catalyst, nonapplicable in this instance. It is clear that the advancement of catalytic activity provided by Pd_5%_/Al_2_O_3_ is a superior material to be used in the more severe second-stage aromatic hydrogenation reactions. The results demonstrate that the adsorption of tetralin is not a limitation with the abundance of palladium species on Pd_5%_/Al_2_O_3_ under the reaction conditions used in this study. This therefore accelerates both cis and *trans*-decalin formation and contradicts conventional *k*_1_ and *k*_2_ relationship assumptions. In addition, the calculated *k*_2_ value is several orders of magnitude greater than the *k*_2_ values for the lower concentration Pd species. The anticipated change in reaction rate constant follows a linear path in response to an increase in Pd concentration, given that the activation energy of Pd over alumina species is expected to be constant. As a result, it is possible that the greater coverage of Pd over alumina which has generated a higher density of Pd clusters on the support, as seen in Figure 5c derived from the EDS analysis, is impacting the electronic interactions on the surface of the catalyst. Coupled with the spherelike morphology which generates a higher quantity of edge sites, exhibited by the Pd_5%_/Al_2_O_3_, demonstrated in Figure 4f with a higher average pore size than the other catalysts demonstrated in Table 2; this has potentially provided the opportunity for tetralin to migrate to and bond with the active centers more readily. Competing against initial naphthalene molecules, tetralin can take advantage of greater dissociative adsorption for hydrogen activation, and subsequent conversion to decalin, as observed previously in Yu et al.^18^ As a result, it is suggested that tetralin to decalin conversions is not structure-sensitive in the presence of Pd_5%_ dispersed over Al_2_O_3_.

While this confirms the advantage of a noble metal-enriched catalytic support in aromatic hydrogenation applications, the high cost and poor sulfur tolerance remains a significant drawback, particularly when dealing with sulfur-rich feeds typically observed in heavier oils. That said, when using Pd_1%_/Al_2_O_3_, the hydrogenation reaction proceeds at a much poorer rate with poor naphthalene conversion to tetralin, in addition to tetralin conversion to cis and *trans*-decalin, as shown in Figure 7c. The *k*_1_ and *k*_2_ values are 1 and 2 orders of magnitude lower than *k*_1_ and *k*_2_ for Pd_5%_/Al_2_O_3_, respectively.

In conventional catalytic regimes, the naphthalene to tetralin conversion is several orders of magnitude greater than the conversion of tetralin to the decalin isomers.^23^ It has been suggested previously that when using a NiMo/Al_2_O_3_ catalyst, the strong adsorption of naphthalene on the active centers inhibits tetralin conversion to decalin until the naphthalene has been completely converted.^42^ The Mo-MMO catalyst broadly concedes to this convention where after 120 min all of the naphthalene has been converted to tetralin, whereas tetralin has been unable to undergo complete conversion to decalin species, as observed in Figure 7e. However, it is noted that in this study, with the exception of NiMo/Al_2_O_3_ where an insignificant conversion of tetralin to decalin occurs, tetralin hydrogenation is simultaneously produced before naphthalene hydrogenation is completed. Accordingly, the data highlights *k*_1_ values as an order greater than the *k*_2_ values. As a result, the mechanism suggested by Su et al.^42^ can be expanded upon. A distinctive difference in aromaticity prevalent between naphthalene and tetralin compounds results in a clear deviation to the hydrogenation reactivity when using a nickel-based catalyst. Tetralin exhibits a greater Pi-electron density than naphthalene which consequently generates a higher aromatic ring resonance energy. This higher energy inhibits hydrogenation reactivity when using non-noble metal species, leading to a strong discrepancy between *k*_1_ and *k*_2_ values. Furthermore, the difference in hydrogenation mechanisms of naphthalene and tetralin has been studied previously when in the presence of a Ni/Al_2_O_3_ catalyst.^12^ While the weak aromaticity of naphthalene accommodates its conversion to tetralin under Pi/sigma adsorption, which demands a single active site, the conversion of tetralin occurs through the Pi-adsorbed species, due its more potent aromaticity. It has previously been found that when using a Ni/Al_2_O_3_ catalyst, multiple Ni atoms are required to generate a tetralin hydrogenation active site, and as a result, the tetralin molecule can be considered catalyst structure-sensitive.^12^ It may therefore be expected that the homogeneous spread of Ni and Al in the MMO may promote more hydrogenation of naphthalene on the molybdenum oxide slabs, as compared to the NiMo/Al_2_O_3_ catalyst. A high conversion of naphthalene could then be responsible for the availability of the active sites to accommodate tetralin conversion, whereas in the NiMo/Al_2_O_3_ catalyst, the reduced dispersion due to the far lower concentration of the nickel promotor, as observed in the EDS analysis Figure 5f, will generate more isolated metallic active sites forming a bottle-neck in the reaction, leading to a reduced tetralin conversion and very limited decalin formation as observed in Figure 6, with a total decalin yield of 1.2%. While the textural characteristics of NiMo/Al_2_O_3_ are more superior to the Ni-LDH-derived Mo species, as would be expected given the favorable properties of an alumina support, it is clear that the abundance and distribution of Ni, observed in Table 1 and Figure 5d,f, respectively, is the defining property in the hydrogenation reaction progression. It is evident that the Mo-MMO catalyst is more comparable to Pd_2%_/alumina, the latter’s reaction progression of which is shown in Figure 7b, with a final yield of 29.0%. Comparatively, the activity of NiMo/Al_2_O_3_ is more comparable to Pd_1%_/Al_2_O_3_, though it generates less tetralin and decalin than the Pd_1%_/Al_2_O_3_ catalyst.

In a previous study, it was concluded that the addition of a basic site-enriched catalyst support may augment the hydrogenation of naphthalene and tetralin, until a certain basic site concentration is reached whereupon the benefits are negated.^13^ It is clear from the activity of tetralin conversion, variation in support materials which leads to variations in the electronic configuration of the catalyst, that a distinct mechanism for the transformation of tetralin into its hydrogenated products is apparent between the catalyst regimes. To highlight these differences in catalytic activity and preferential pathways for tetralin hydrogenation, plots of *cis*/*trans*-decalin vs concentration of decalin formed were analyzed.

### 3.3 *Cis*/*trans*-decalin Ratio

**Figure 9:**
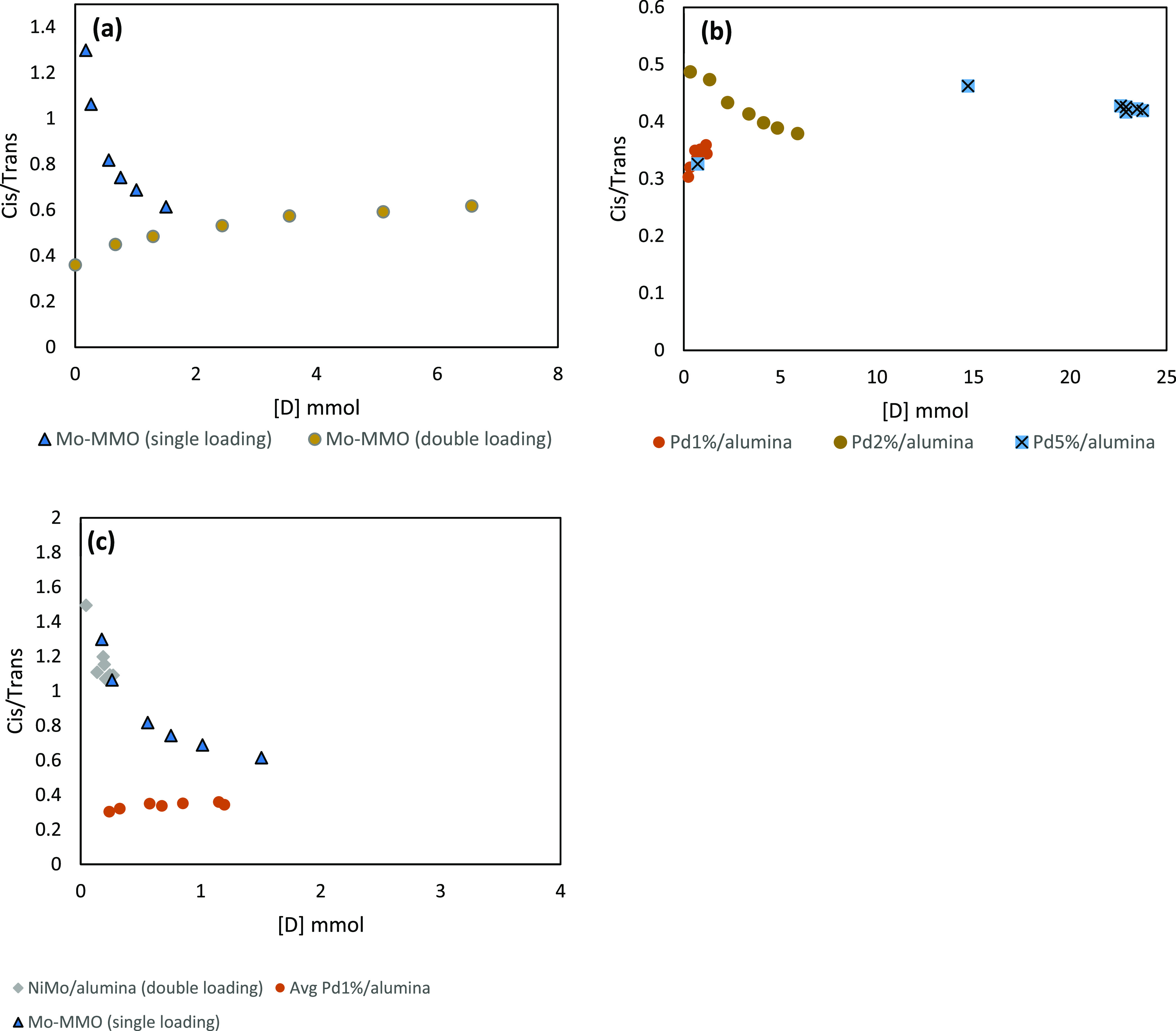
Plots of *cis/trans* ratio against decalin concentration for (a) two separate catalyst loadings of Mo-MMO and (b) including Pd_5%_/alumina, Pd_2%_/alumina, and Pd_1%_/alumina and (c) NiMo/alumina, Pd-MMO, Mo-MMO (single loading), and Pd_1%_/alumina.

It is observed in Figure 9a that when the Mo-MMO catalyst is used with the same catalyst to reactant ratio as the palladium-based catalysts, the *cis/trans* ratio begins at a comparatively high level compared to the other catalysts shown in both Figure 9a,b, at approximately 1.30. It begins to decline significantly as tetralin conversion to decalin ensues. However, when the basic site-enriched Mo-MMO support catalyst to reactant ratio is doubled to Mo-MMO double loading, for the same concentration of decalin, a greater concentration of *trans*-decalin is apparent initially with a cis/trans ratio of 0.32, and the trend is reversed leading to a gradual increase to 0.68 as the tetralin is converted. When using the acidic Al_2_O_3_-supported NiMo catalyst (used with double loading only), as shown in Figure 9c, the cis/trans ratio begins at 1.49 and gradually decreases with tetralin conversion to decalin, reaching a final ratio of 1.09. This trend is similar to that of the Mo-MMO single loading at the early tetralin conversion stage. When comparing the double loadings of NiMo/Al_2_O_3_ and Mo-MMO catalysts, the disparity between cis/trans ratio is clear. While the reaction has not proceeded to the same extent, it is clear that early conversion of the double loading Mo-MMO yields an enriched *trans*-decalin product in direct contradiction to NiMo/Al_2_O_3_. However, as tetralin conversion to decalin increases, so the concentration ratio appears to converge.

It has been postulated that the concentration of both the *cis* and *trans* isomers is dependent on two factors: (i) isomerization activity of the catalyst and (ii) decalin–tetralin adsorption competition.^15^ At low conversion of tetralin, the basic sites on the Mo-MMO catalyst are responsible for promoting isomerization to *trans*-decalin. There is however a significant reduction in *cis* isomerization to *trans*-decalin as the tetralin concentration decreases, after which the conversion stabilizes resulting in a *trans*-depleted decalin product with a 0.62 *cis/trans* ratio. The observed results agree with Rautanen et al.,^12^ wherein long experiments resulting in poor tetralin conversion have resulted in an increase in *cis*-decalin. This is exemplified with the Mo-MMO single loading and NiMo/Al_2_O_3_, observed in Figure 9c, wherein as the tetralin conversion slowly increases, rapid *cis* to *trans* isomerization takes place, particularly in the case of NiMo/Al_2_O_3_.

Furthermore, increases in competition for adsorption sites could greatly reduce the occurrence of the *cis*-*trans* isomerization reaction.^15,43^ This is because although it has been previously reported that the *cis* to *trans* isomerization has a higher rate constant than tetralin hydrogenation, the tetralin molecules form a rigid parallel morphology against the surface of the catalyst compared to the hinged *cis*-decalin molecule.^15^ This may explain the disparity between the single and double loadings of Mo-MMO, whereupon increasing the availability of adsorption sites, an increase in the cis to trans isomerization activity was observed, i.e., the presence of tetralin was unable to block the active sites. This is also supported by the enhanced activity of *cis* to *trans* isomerization of Ni compared to Pd.^15^

There is a clear difference between nickel and molybdenum-containing catalysts and palladium bearing catalysts. With the latter, at both high concentration and low concentration on Al_2_O_3_, with both high and low conversion to decalin, the cis/trans ratio remains in favor of *trans*-decalin, as highlighted in Figure 9b. The ratios for Pd_1%_, Pd_2%_, and Pd_5%_ end with 0.34, 0.38, and 0.42, respectively. This would suggest that there is a clear tendency to produce the *trans*-isomer under palladium irrespective of tetralin concentration. This corroborates with previous research which has shown that palladium occupies a higher *trans*-selectivity than its nickel-containing catalyst counterparts.^12,15^ The comparative enrichment of *trans*-decalin, however, has been attributed to a very low intrinsic isomerization activity on the palladium catalyst which essentially stabilizes the produced *cis/trans* ratio throughout the entire tetralin conversion progression.^15^

An underlying mechanism has been suggested by Dokjampa et al.,^15^ which speculates that the cis/trans ratio is reliant on the orientation of the Δ^1,9^-octalin intermediate. Hydrogen incorporation into this octalin species is especially fast in the case of nickel catalysts, while the palladium catalyst accommodates a roll over on the surface, culminating in an orientation wherein the H atom in position 10 of the ring is facing away from the surface. This can be inferred considering the conformation of Δ^1,9^-octalin on the catalyst surfaces; the syn nature of hydrogen addition requires the addition of H atoms to a double bond from the same side. Moreover, while the Δ^9,10^-octalin exclusively produces cis decalin, the greater hydrogenation rate of Δ^1,9^-octalin over noble metal catalysts, with the 10 atoms facing away from the catalytic surface, greatly reduces isomerization to Δ^9,10^-octalin resulting in a greater amount of *trans*-decalin production.^12^

When using nickel and molybdenum catalysts, it is stipulated that the greater initial concentration of cis relative to trans is indicative of a preferred orientation of Δ^1,9^-octalin, wherein the hydrogen atom in position 10 faces toward the surface. The addition of hydrogen atoms to positions 1 and 9 occurs on the same side, thereby producing *cis*-decalin.^15^

## 4 Conclusions

Mixed metal oxide carriers bearing a 3.3:1 Ni/Al ratio were synthesized from anionic clay species. The incipient wetness impregnation method was used to deposit molybdenum onto the surface of the support. The catalytic materials were tested in liquid-phase naphthalene hydrogenation at 250 °C and 40 bar H_2_. When comparing Ni and Mo catalysts, the higher the loading of Ni present over the catalyst, the higher the conversion of naphthalene. It is also evident that when contrasting different Pd loadings over alumina, a higher loading of Pd produces a higher yield of decalin with the total yield ranging from 99.5 to 5.9% conversion for Pd_5%_/Al_2_O_3_ and Pd_1%_/Al_2_O_3_, respectively. Reaction rate constants were derived from a pseudo-first-order kinetic pathway describing naphthalene to tetralin (*k*_1_) and tetralin to decalin (*k*_2_) hydrogenation. The Mo-MMO catalyst achieved comparable reaction rates to Pd_2%_/Al_2_O_3_ at double concentration, exceeding Pd_1%_/Al_2_O_3_, and NiMo/Al_2_O_3_. When using Pd_5%_/Al_2_O_3_, tetralin hydrogenation was favored over naphthalene hydrogenation culminating in a *k*_2_ value of 0.224 compared to a *k*_1_ value of 0.069, detouring from the conventional assumption that *k*_1_ ≫ *k*_2_. The impact of catalyst on the cis/trans ratio against decalin concentration is clear. When using the Mo-MMO catalyst, the products initially contain a comparatively low *cis*-decalin concentration, followed by an inhibition of cis to trans isomerization and therefore a progressively more *cis*-decalin enriched product, mirroring that of the NiMo/Al_2_O_3_ catalyst. In comparison, Pd_1–5%_/Al_2_O_3_ catalysts generate a much lower cis/trans ratio irrespective of tetralin concentration and palladium abundance, highlighting preferential for *cis*- to *trans*-decalin isomerization activity over Pd active sites. Consequently, NiMo-bearing catalysts, although offering poorer performance, may provide greater advantages in simultaneous HYD and HDA processes due to *cis*-decalin’s greater selectivity to indanes and alkyl-cyclohexanes. It is also evident that the enriched Mo-MMO offers benefits over the conventional refinery catalyst due to the comparative ease of synthesis of the LDH material in addition to more economic methods of formulation. Future work should include both the impact of sulfur on the hydrogenation process and catalyst performance, as well as hydrodecyclization of the *cis*-decalin product to yield alkyl naphthenes.
